# Prehistoric cooking versus accurate palaeotemperature records in shell midden constituents

**DOI:** 10.1038/s41598-017-03715-8

**Published:** 2017-06-15

**Authors:** Peter Müller, Philip T. Staudigel, Sean T. Murray, Robert Vernet, Jean-Paul Barusseau, Hildegard Westphal, Peter K. Swart

**Affiliations:** 1 Leibniz Centre for Tropical Marine Research, 28359 Bremen, Germany; 2 Rosenstiel School of Marine and Atmospheric Science, University of Miami, Miami, FL 33149 USA; 3 Institut Mauritanien de Recherches Scientifiques, Nouakchott, Mauritania; 4 Centre de Formation et de Recherche sur les Environnements Méditerranéens, University of Perpignan Via Domitia, Perpignan, France; 5 Department of Geosciences, University of Bremen, Bremen, Germany

## Abstract

The reconstruction of pre-depositional cooking treatments used by prehistoric coastal populations for processing aquatic faunal resources is often difficult in archaeological shell midden assemblages. Besides limiting our knowledge of various social, cultural, economic and technological aspects of shell midden formation, unknown pre-depositional cooking techniques can also introduce large errors in palaeoclimate reconstructions as they can considerably alter the geochemical proxy signatures in calcareous skeletal structures such as bivalve shells or fish otoliths. Based on experimental and archaeological data, we show that carbonate clumped-isotope thermometry can be used to detect and reconstruct prehistoric processing methods in skeletal aragonite from archaeological shell midden assemblages. Given the temperature-dependent re-equilibration of clumped isotopes in aragonitic carbonates, this allows specific processing, cooking or trash dispersal strategies such as boiling, roasting, or burning to be differentiated. Besides permitting the detailed reconstruction of cultural or technological aspects of shell midden formation, this also allows erroneous palaeoclimate reconstructions to be avoided as all aragonitic shells subjected to pre-historic cooking methods show a clear alteration of their initial oxygen isotopic composition.

## Introduction

Many prehistoric coastal populations have exploited the faunal resources of adjacent aquatic environments^1, 2^. The remains of molluscs (shells) and fish (otoliths) were often deposited in the vicinity of prehistoric settlements forming mounds known as shell middens. The composition, internal structure or the spatial and temporal distribution of these shell middens represent a valuable source of information about human dispersal, site-specific occupation pattern, subsistence strategies, associated dietary preferences or fishing and foraging seasonality^3, 4^. Moreover, mollusc shells or fish otoliths derived from shell middens can provide proxy records such as oxygen isotopes which enable the reconstruction of highest-resolution (sub-seasonal) palaeoenvironmental conditions of ancient coastal environments^5–7^. Despite allowing the reconstruction of foraging seasonality^3, 4^, past upwelling intensities^8–10^ or seasonal freshwater discharge patterns^11–13^, ontogenetic geochemical signatures of shell midden constituents have become an important tool for the reconstruction of ancient water temperature seasonality within coastal areas^14–17^. In particular shell midden deposits which cover time spans of up to several millennia allow the long-term reconstruction of environmental change with a sub-seasonal resolution^18^.

As subsistence was the primary reason for exploiting aquatic faunal resources for many prehistoric cultures, most shell midden constituents were most-likely subjected to certain processing or cooking techniques prior to deposition^4^, In particular molluscs, where the comparably small amount of edible flesh is protected by tightly closed valves or an operculum require sophisticated processing strategies in order to efficiently extract the small edible portion^4, 6^. However, evidence for certain processing or cooking techniques such as pottery and tool fragments or blackened/burned stones are often sparse in shell midden deposits or allow a variety of potential interpretations regarding specific cooking or processing strategies. This is particularly true at transitory preparation sites near fishing or shellfish gathering grounds rather than close to prehistoric settlements.

While usually unknown processing methods limit our understanding of shell midden formation, processing or cooking methods can alter the geochemical proxy records of calcareous skeletal remains, potentially introducing large uncertainties into palaeoclimate reconstructions^19–21^. Hence, the reliable reconstruction of prehistoric processing methods is crucial in order to avoid erroneous palaeoclimate reconstructions based on geochemical proxies in shell midden constituents.

In this study, we tested the potential application of carbonate clumped-isotope thermometry on aragonitic mollusc shells and fish otoliths to reconstruct specific prehistoric cooking methods. Carbonate clumped-isotope thermometry is based on the preferential formation of ^13^C-^18^O bonds (“clumping”, expressed as Δ_47_) in the carbonate molecule with decreasing carbonate formation temperature, independent of the isotopic composition of the precipitating fluid^22–24^ and therefore circumvents uncertainties in conventional oxygen-isotope thermometry which commonly derive from unknown ancient water chemistry^23–25^. In addition, clumped-isotope thermometry allows reconstructing the thermal histories of carbonate rocks as the relative abundance of ^13^C-^18^O bonds can be re-equilibrated during post-formational heating^26, 27^. The rate and magnitude of ^13^C-^18^O bond re-ordering is a function of temperature and exposure time and can occur even in closed systems without any isotope exchange with ambient media^26–28^. While re-equilibration in calcite occurs over geological time scales, aragonite shows much higher re-ordering rates resetting the clumped-isotopic signature within minutes to hours^29^. Thus, clumped-isotope thermometry might represent an ideal tool to detect pre-depositional heating of aragonitic shell midden constituents and may allow certain processing or cooking practices to be differentiated.

In order to test this hypothesis, we exposed the right valves of modern hard clams (*Mercenaria campechiensis*, Gmelin 1791) cultured in the Gulf of Mexico to a variety of prehistoric cooking methods. Our experiment comprised untreated shells (corresponding to manually opened shells and serving as controls), shells boiled in seawater (~100 °C), roasted over charcoal (174 ± 13 °C) and directly burned in charcoal (559 ± 95 °C). For all treatments, exposure times ranged from 15 minutes to 6 hours. Afterwards, we measured the bulk clumped-isotopic composition (Δ_47_), oxygen and carbon isotope values (δ^18^O and δ^13^C, respectively) as well as the potential conversion of the primary aragonite into secondary calcite.

In addition, we measured bulk δ^18^O and Δ_47_ values of mid-Holocene shell midden constituents excavated in the northern part of the Mauritanian coast (Banc d’Arguin), radiocarbon dated to 5,020–5,320 cal. yrs. BP. These samples comprised four catfish otoliths (*Carlarius* spp.) and seven bivalve shells (*Senilia senilis*, Linnaeus 1758). We also analysed additional samples drilled from the hinge region of each bivalve shell, as well as high-resolution ontogenetic oxygen-isotope records of three exemplary shells.

## Results

### Experimental shells

**Figure 1:**
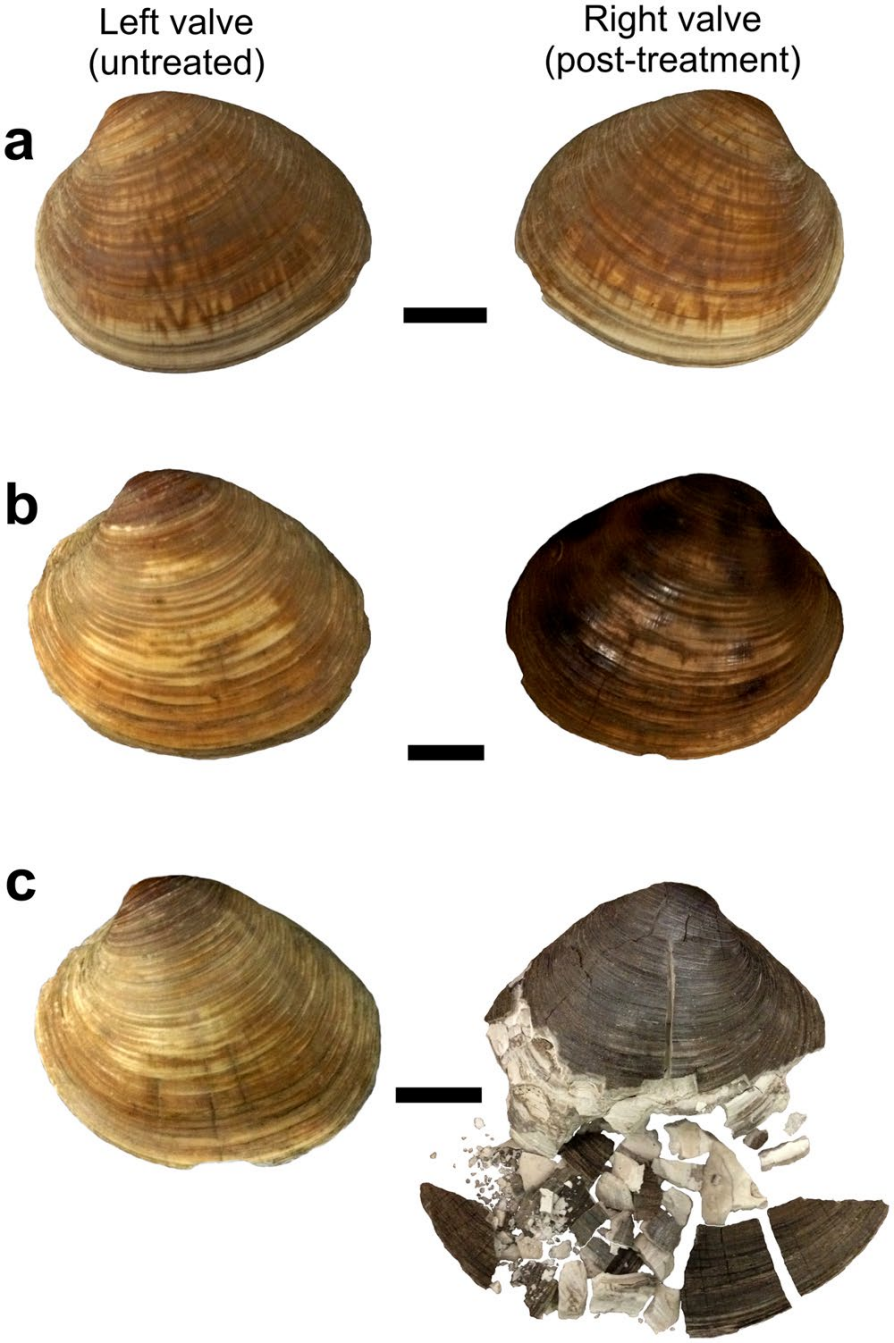
Visual comparison of untreated left valves and the corresponding right valves exposed to the different treatments prior to hydrogen peroxide cleaning. (**a**) Boiling for 40 minutes. (**b**) Roasting for 240 minutes. (**c**) Burning for 240 minutes. Scale bars represent 1.0 cm.

**Figure 2:**
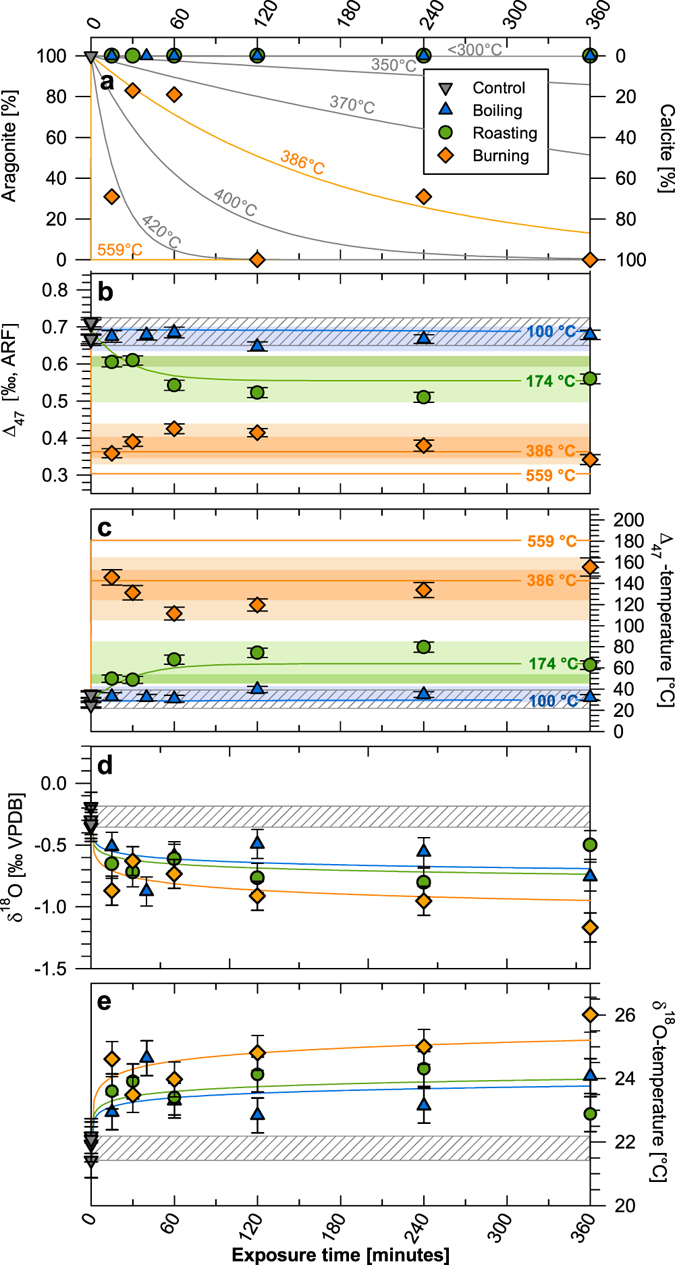
Alteration of mineralogy and geochemical proxies in modern *Mercenaria campechiensis* shells by simulated prehistoric cooking methods: Untreated (grey triangles and grey striped areas), boiled (blue triangles), roasted (green circles), and burned shells (orange diamonds). (**a**) Conversion of primary aragonite into secondary calcite with the predicted aragonite-calcite conversion based on the Arrhenius model for biogenic aragonite of Staudigel & Swart^29^ (solid lines). (**b**) Clumped-isotopic composition (Δ_47_) reported in the absolute reference frame (ARF) defined by Dennis *et al*.^24^ of the bivalve shells after exposure to the different cooking methods. Solid lines represent predicted Δ_47_ values using the Arrhenius model of Staudigel & Swart^29^. Error bars represent mean standard errors of the individual measurements. The grey striped area represents the range of untreated control shells (n = 5). Shaded areas represent the characteristic Δ_47_ windows for each cooking treatment over the entire experiment duration (light) and realistic cooking durations of <30 minutes (dark). (**c**) Calculated Δ_47_-based water temperature estimated using the equation of Dennis *et al*.^24^ and corresponding theoretical Δ_47_-based water temperature estimates predicted by the thermodynamic model of Staudigel & Swart^29^. Similar to panel (b), light and dark blue/yellow areas represent the characteristic Δ_47_-windows translated into water temperature. (**d**) Change in δ^18^O_Shell_ over time of exposure for the different cooking methods. Solid lines represent exponentially fitted curves highlighting the overall trend towards lower δ^18^O_Shell_ values. (**e**) Calculated δ^18^O_Shell_-based water temperatures using the equation of Grossman & Ku^30^ and assuming a constant δ^18^O_Seawater_ of 0.0‰ VSMOW.

Depending upon the cooking temperature, variable changes in shell mineralogy, visual appearance and outer shell structure occurred within the different cooking treatments (Fig. 1). Boiling and roasting of the shells caused bleaching and blackening of the outer organic layer, respectively. While the cooked shells consistently showed a pristine outer shell structure, some roasted shells exhibited small cracks (<0.5 mm) perpendicular to the ventral margin. Bleaching and blackening of the cooked and roasted shells’ periostracum entirely disappeared after the removal of the periostracum using hydrogen peroxide. Despite the sporadically occurring cracks, overall shell integrity remained pristine, hindering a clear differentiation from uncooked control shells. Direct burning of the shells resulted in blackening of the outer surface, whitening of the inner shell layer, and was accompanied by an immediate loss of structural integrity and the conversion of the solid shell structure into brittle and chalky fragments. Boiling and roasting did not cause any conversion of initial aragonite into secondary calcite (Fig. 2a), while the burned shells showed a generally increasing conversion of primary aragonite to secondary calcite over the time of exposure.

The exposure of bivalve shells to different cooking practices caused an alteration of their clumped-isotopic composition (Δ_47_). Re-ordering rates in aragonite were high enough to cause an immediate reduction in Δ_47_ where the magnitude is generally a function of cooking temperature and exposure time. The lowest amount of change occurred in the boiling treatment (~100 °C) where the reduction in the Δ_47_ values was not significantly different from the control shells. This agrees with the thermodynamic model for clumped-isotope re-ordering in aragonite at 100 °C^29^ which suggests that an exposure time of several hours to temperatures ~100 °C is not sufficient to cause a significant decrease in Δ_47_ values. In contrast, roasting and burning caused an immediate decrease in Δ_47_ to values ranging from 0.510 to 0.609‰, and 0.342 to 0.425‰, respectively. As shown in Fig. 2b and c, experimental lowering of shell Δ_47_ values within the boiling and roasting treatments are in good agreement with thermodynamic models for the relevant temperatures over the time of exposure^29^.

Independent of cooking temperature or exposure time, all cooking treatments resulted in a statistically significant lowering of the bulk δ^18^O_Shell_ values, reducing their potential for reliable water temperature reconstructions (Fig. 2d and e). The maximum decrease of δ^18^O_Shell_ values was −0.61‰ for boiling, −0.53‰ for roasting, and −0.90‰ for the burning treatment. Using the equation of Grossman & Ku^30^, the decrease in δ^18^O_Carbonate_ values translates into water temperature overestimations of 2.9, 2.5 and 4.2 °C, respectively. While δ^18^O_Carbonate_ values of all treatments were significantly different from the control shells, there was no significant difference between the different cooking treatments. We observed no statistically significant alteration trend with respect to shell δ^13^C_Carbonate_ values among the different treatments. However, the variability in δ^13^C_Carbonate_ values of the treated shells exceeds the range measured in the control shells, indicating that isotope exchange reactions also affected shell δ^13^C_Carbonate_ values.

### Archaeological samples

All mid-Holocene otoliths are mainly composed of aragonite (96–100%) which is in agreement with the initial mineralogy of modern *Carlarius* spp. otoliths (~98% aragonite, 2% calcite for *Carlarius heudelotii*). Likewise, all mid-Holocene *S*. *senilis* shells were composed of 100% aragonite which is the initial mineralogy also found in modern shells^31^. From the mineralogical perspective, the archaeological samples can therefore be considered pristine. All mineralogical data are provided in Supplementary Tab. 4.

Mid-Holocene otolith Δ_47_-values (0.667 to 0.716‰) largely agree with predicted values based on modern water temperature observations^31, 32^. In contrast, mid-Holocene bivalve shells show highly variable Δ_47_ values ranging from 0.601 to 0.716‰ which is considerably lower than expected for natural environments. Noteworthy, bulk samples of the shells show higher variations in Δ_47_ and tend towards lower Δ_47_ values (0.601 to 0.716‰) than corresponding samples from the hinge region of the same individuals (0.651 to 0.697‰). Subsequently, Δ_47_-based water temperature reconstructions using the bulk shell samples result in unreasonably high palaeo-water temperature estimates (23.9 to 51.0 °C). In contrast, the samples drilled from the hinge region of the same shells provide more realistic water temperature estimates for coastal intertidal environments (28.0 to 38.3 °C).

**Figure 3:**
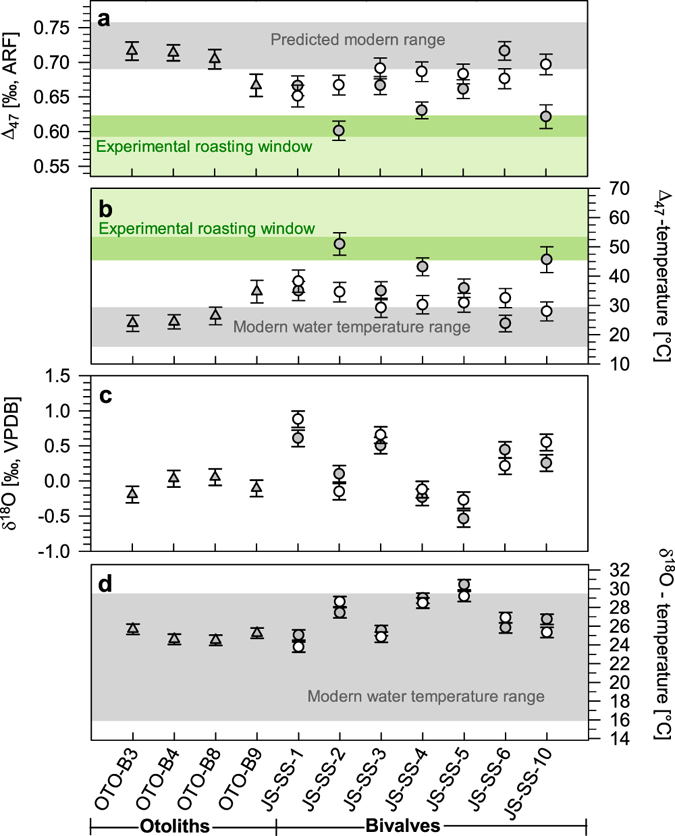
Clumped and oxygen isotopic composition with corresponding water temperature estimates of mid-Holocene fish otoliths and bivalve shells. (**a**) Clumped-isotopic composition (Δ_47_) reported in the absolute reference frame (ARF) defined by Dennis *et al*.^24^ of bulk otoliths (grey triangles), bulk bivalve shells (grey circles) and hinge samples from the bivalve shells (open circles). Error bars represent mean standard errors of the individual measurements. The grey area represents the theoretical Δ_47_-range based on modern local water temperature data^31^ translated into Δ_47_-values using the equation of Dennis *et al*.^24^. The shaded green area represent the experimentally determined Δ_47_-window for roasting at 174 ± 13 °C for 6 hours (light green) and realistic cooking duration of >30 minutes (dark green). (**b**) Δ_47_-based water temperature reconstruction using the equation of Dennis *et al*.^24^ with modern water temperature data^31^. Shaded green areas represent the experimentally determined Δ_47_-window of the roasting treatment translated into water temperature for 6 hours (light green) and realistic cooking duration of >30 minutes (dark green). (**c**) Oxygen isotopic composition of bulk fish otoliths, bulk bivalve shells and hinge samples from the bivalve shells. (**d**) Oxygen-isotope-based water temperature reconstructions for the otoliths using the equation of Thorrold *et al*.^33^ assuming a constant δ^18^O_Seawater_ value of +0.68‰ VSMOW^32^ and oxygen-isotope based water temperature reconstructions for the bivalve shell samples using the equation of Grossman & Ku^30^ assuming a constant modern δ^18^O_Seawater_ value of +1.57‰ VSMOW^31^ with the modern local water temperature range^31^.

Mid-Holocene bulk δ^18^O_Carbonate_ values are consistent amongst all otoliths and bivalve shells, ranging from −0.54 to +0.88‰ VPDB (Fig. 3). Assuming mid-Holocene δ^18^O_Seawater_ values of +0.68 and +1.57‰ VSMOW for the otoliths^32^ and bivalve shells^31^, respectively, reconstructed water temperatures are slightly higher during the mid-Holocene than observed today. Measured ontogenetic bivalve δ^18^O records show a clear sinusoidal cyclicity that covers three to six years of growth. Measured ontogenetic δ^18^O_Carbonate_ values range from −1.8 to +1.5‰ VPDB, translating into mid-Holocene water temperatures ranging from 21.1 to 36.2 °C.

## Discussion

### Experimental data

Previous studies on the effect of prehistoric cooking on mollusc shells simulated cooking temperatures ranging from 100 to 500 °C^20, 21^. Our mineralogical data agrees with these studies as they also showed a conversion of primary aragonite into secondary calcite at temperatures >300 °C. This is consistent with other experimental studies that show temperatures between 300–400 °C as the minimum temperature for the conversion of biogenic aragonite into calcite^29, 34, 35^. However, the burned shells show a heterogeneous and considerably lower conversion of the primary aragonite into secondary calcite than predicted by the Arrhenius model of Staudigel & Swart^29^ for 559 °C where aragonite should entirely convert into calcite within less than a second. The lowest burning temperatures measured within our experiment (≥386 °C, see Supplemental Fig. 1) falls exactly in the window where only a partial conversion of aragonite into calcite would be expected for the experimental exposure times (see Fig. 2a)^29^. We therefore presume that the heterogeneous conversion among the burned shells can be related to the spatial and temporal temperature variability experienced by the individual shells throughout the experiment (see Fig. 2a and Supplemental Fig. 1).

Based on their experimental results, Milano *et al*.^20^ proposed the use of crystal microstructure for the detection of pre-depositional heating in shell midden deposits which has also been used in other previous studies on diagenetic alteration of shell midden constituents^36^. However, Milano *et al*.^20^ only observed changes in crystal microstructure associated with conversion of primary aragonite into secondary calcite. Since we did not find any conversion within the boiling and roasting treatments, our data indicate that mineralogical^37^ or shell microstructure analyses^20, 36^ may fail to detect pre-depositional heating if cooking temperatures are below the aragonite-calcite conversion window of 300–400 °C. Although burning causes a clear conversion of the primary aragonite into secondary calcite and would therefore be easily identifiable in shell midden deposits, the preservation potential of small burned shells in shell midden deposits is questionable considering their brittle shell structure. However, as the breakage of the burned shells occurred predominantly along the ventral margin, the thicker hinge region might still be preserved in shell midden deposits, in particular for larger shell specimens.

The observed minor overshooting of theoretical Δ_47_ values by the roasting treatment was most likely caused by short-term temperature spikes (>200 °C) which were also measured during the experiment (see Supplemental Fig. 1). In contrast, the observed undershooting of theoretical equilibration temperatures by the burning treatment could have been caused by several different mechanisms including: (1) disorder-to-order re-equilibration during cooling to ambient air temperature after the experiment, (2) complex interplays of re-ordering processes as a consequence of aragonite/calcite transitions during the experiments or (3) spatial and temporal temperature variability of the burning treatment including potential temperature gradients between the charcoal surface and the shells. It appears unlikely that cooling to ambient air temperature caused the observed underestimation of temperature as disorder-to-order re-equilibration occurs over considerably longer time scales^28^. Instead, we suspect that the conversion of aragonite into calcite causes the deviation from the thermodynamic model which has also been shown by the experiments of Stolper & Eiler^35^ and Piasecki^38^, involving at least three different mechanisms of clumped-isotope re-ordering during aragonite-calcite conversion. These mechanisms are (1) a sharp decrease in Δ_47_ during the initial heating of the primary aragonite (annealing) followed by a (2) partial conversion of aragonite into calcite causing a re-increase in Δ_47_ and (3) a second re-ordering phase of the secondary calcite resulting in a decrease in Δ_47_ values with considerably lower re-equilibration rates. This complex model of clumped-isotope re-ordering during the transition of aragonite into calcite is supported by our data because the measured trajectory of Δ_47_ alteration within the burning treatment exactly follows the pattern described by Piasecki^38^. Assuming a lower treatment temperature of only 386 °C increases the goodness of fit between the measured and predicted Δ_47_ values of the burning treatment (386 versus 559 °C models shown in Fig. 2b and c). Thus, lower burning temperatures of some shells might explain the overall lower alteration magnitude found in this study compared to the Arrhenius model of Staudigel & Swart^29^ for 559 °C (Fig. 2b and c). However, our experiment was designed to simulate (spatially and temporarily heterogeneous) prehistoric cooking practices and has thus only limited validity regarding the underlying thermodynamic mechanisms of clumped-isotope re-ordering during the aragonite/calcite transition.

Regardless of experimental uncertainties, the lower Δ_47_ values of the shells in the roasting and burning treatments are significantly different from the control as well as from each other. In particular, if only shells heated for less than 30 minutes are considered, the decreases in Δ_47_ values result in a confined Δ_47_/temperature window for each cooking treatment. Thus, our experimental data strongly support the aforementioned hypothesis that clumped-isotope thermometry applied on aragonitic skeletal structures represents a suitable diagnostic tool for detecting and differentiating between certain prehistoric cooking techniques in shell midden deposits.

The measured alterations of δ^18^O_Carbonate_ values generally agree with previous studies in terms of magnitude, however, they differ in the minimum temperature required for a measurable alteration of oxygen isotopes in aragonitic skeletal structures by prehistoric cooking practices. Andrus & Crowe^19^ reported no alteration of δ^18^O_Otolith_ values at temperatures up to ~200 °C for fish otoliths, and only detected changes in isotopic signatures of otoliths burned directly in hot (~800 °C) coals^19^. In contrast, Milano *et al*.^20^ showed only a clear decrease in the δ^18^O_Shell_ values of mollusc shells at temperatures of >300 °C. We suggest that the lower minimum temperature for alteration of oxygen isotopes in aragonitic skeletal structures found in this study as well as by Milano *et al*.^20^ compared to that of Andrus & Crowe^19^ is related to the direct exposure of the aragonitic mollusc shells to the cooking medium. Andrus & Crowe^19^ exposed the entire fish to the different cooking treatments with otoliths protected from direct heat inside the vestibular system. This hypothesis is supported by the general agreement between our data and those data of Milano *et al*.^20^ who also observed a similar decrease in the δ^18^O values of mollusc shells at temperatures between 300 and 700 °C. The experiment of Milano *et al*.^20^, however, did not include relevant roasting temperatures between 100 and 300 °C which most likely represent the temperature window where a decrease in the δ^18^O_Carbonate_ values occurs without a conversion of primary aragonite into secondary calcite.

The different magnitudes of δ^18^O_Shell_ alteration between this study and Milano *et al*.^20^ at lower temperatures (<300 °C) indicates that experimental design plays a key-role in assessing the impact of prehistoric cooking practices on palaeoclimate proxy signatures of shell midden constituents. While controlled oven experiments are a suitable approach for studying the underlying processes of aragonite-calcite conversion and identifying isotope-exchange mechanisms, the realistic simulation of prehistoric cooking practices using coal/wood fire provides more reliable estimates regarding the absolute alteration of palaeoenvironmental proxies in shell midden constituents. The stronger alteration at lower temperatures found in this study is probably related to the presence of water in the boiling treatment, high-frequency temperature variability in the roasting and burning experiment, and higher CO_2_ and CO concentrations and fluxes compared to the closed oven setups of Milano *et al*.^20^. Such factors are likely to facilitate isotope-exchange reactions between the carbonate mineral and the ambient cooking medium and can thus explain the more extensive alteration found in this study with respect to δ^18^O_Carbonate_ values at lower cooking temperatures. Together with temperature fluctuations of realistic roasting and burning experiments, such processes might also explain the observed non-linearity of δ^18^O alteration within the different treatments. However, experimentally determined alteration during prehistoric cooking will most likely also vary depending on the flame temperatures or different wood types used in the experiments.

### Archaeological data

Although absolute mid-Holocene clumped-isotope-based water temperature reconstructions might be imprecise as a result of small but unknown deviation from theoretical equilibrium precipitation (i.e. “vital effects”), measured Δ_47_ values of the mid-Holocene bivalve shells are considerably lower than expected for natural environments. While our experimental data suggests that these shells were heated prior to deposition, their pristine aragonitic mineralogy suggests that they did not experience temperatures higher than 300–400 °C. The overlap of the measured Δ_47_ values of the mid-Holocene shell with our experimental data obtained from the roasting treatment (see Fig. 3) indicates cooking temperatures in the range of roasting or fumigation. The difference in Δ_47_ values between the bulk and the hinge samples of the same bivalve shells suggests that the outer shell surface of most shells experienced considerably higher temperatures than the corresponding hinge region. Therefore, we presume that the articulated shells were placed on heated surfaces to cause heat-related muscle relaxation which predominantly affected the outer shell surface and which represents a well-known processing technique for bivalve shells reported in many ethnographic observations^4^. This technique would imply that not all excavated valves should show heat-related Δ_47_ alterations (i.e. only the lower valve), which is also supported by our data (Fig. 3).

An alternative explanation for the post-mortem alteration of clumped-isotopic signatures in the mid-Holocene bivalve shells might be the long-term sub-aerial exposure with increased soil temperatures as a consequence of high solar insolation in this sub-Saharan region. However, assuming a potential re-equilibration due to surface exposure throughout the Holocene, one would also expect different bivalve shells to exhibit the same degree of alteration, similar Δ_47_ values within individual shells (hinge versus bulk samples), as well as similar Δ_47_ values for the otoliths and bivalves excavated from the same shell midden. Thus, it appears unlikely to us that the exposure to increased sediment surface temperatures caused the observed trend in the clumped-isotopic signature of the mid-Holocene samples.

**Figure 4:**
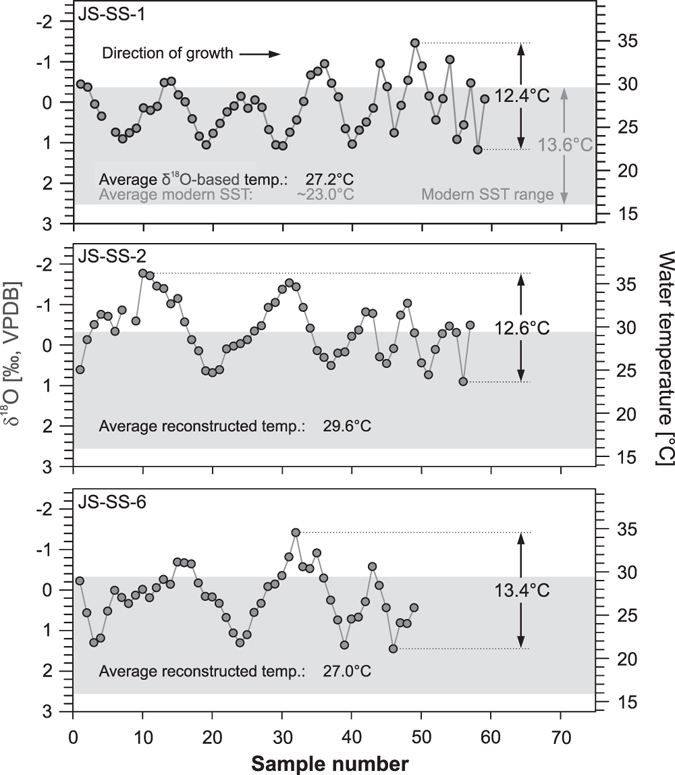
Ontogenetic oxygen-isotope records of three archaeological mid-Holocene *Senilia senilis* shells and comparison with modern coastal sea surface temperature variability of the eastern Banc d’Arguin^31^. Water temperature reconstruction was done using the equation of Grossman & Ku^30^ assuming a δ^18^O_Seawater_ value of +1.57‰ VSMOW^31^. Error bars for the measured δ^18^O_Carbonate_ values and related water temperature estimates are smaller than the symbol size.

Although the clumped-isotopic signatures of the bivalve shells strongly suggests pre-depositional heating, the bulk oxygen isotopic composition provides slightly higher, but still reasonable, water temperature estimates for coastal regions of NW Africa during the mid-Holocene, potentially indicating only a minor degree of pre-depositional alteration. Similarly, the high-resolution ontogenetic δ^18^O-based water temperature records show a clear sinusoidal oscillation but with on average ~5 °C higher water temperatures during the mid-Holocene than observed today in this region (Fig. 4)^31^. Whether these δ^18^O_Carbonate_ values accurately identify warmer water temperatures during the mid-Holocene or if they overestimate ancient water temperatures due to pre-depositional alteration remains speculative. However, since the sinusoidal shape of the ontogenetic water temperature records seem to be preserved within all mid-Holocene shells and their water temperature amplitudes (12.4, 12.6 and 13.4 °C) agree with the modern values of this region (13.6 °C)^31^, we presume that even if these shells were altered by pre-depositional heating, their internal δ^18^O-range might have been preserved and can still provide information about water temperature seasonality and, for example, the season of capture. However, future studies on the influence of prehistoric cooking on ontogenetic oxygen isotope records in mollusc shells are necessary to further examine the potential alteration of ontogenetic oxygen-isotope records.

#### Implications for palaeoclimate reconstructions

This study strongly suggests that pre-depositional heating of aragonitic shell midden constituents can cause considerable, but most likely unnoticeable, errors in palaeoclimate reconstructions. Depending on the cooking temperature, the alteration of oxygen isotopes in aragonitic shells is rather small (<0.9‰ ≈ <4.2 °C) and occurs even without a clear conversion of primary aragonite into secondary calcite, particularly at cooking temperatures <300 °C. This implies that such pre-depositional alterations are hardly differentiable from natural ancient water temperature variations and that conventional mineralogical analyses might fail to detect such pre-depositional heating. However, our data clearly show that clumped-isotope thermometry enables the reconstruction of the thermal history of aragonitic skeletal structures and therefore helps avoiding erroneous paleoclimate reconstructions introduced by their exposure to considerable heat prior to their deposition.

While additional experiments are needed to fully understand the underlying thermodynamic mechanisms controlling isotope-exchange mechanisms, our data show that a careful assessment of sample preservation including clumped-isotope analysis is necessary in future studies to avoid erroneous paleoclimate reconstructions using aragonitic shell midden constituents. Considering the higher stability of calcite relative to aragonite, it is likely that calcitic mollusc shells may well retain their primary oxygen-isotope ratio during exposure to prehistoric cooking practises although this also needs to be tested in future studies.

#### Implications for shell midden archaeology

As shown in this study, clumped-isotope thermometry represents a unique diagnostic tool for detecting but also differentiating between certain prehistoric cooking practices employing aragonitic shell midden constituents which can provide new insights into various aspects of shell midden formation. Despite evidence of human behaviour (e.g. ritualistic versus nutrition purposes of shellfish foraging or particular subsistence strategies)^39–41^ and information about the function of particular sites (e.g. processing sites, dinner-time camps or residential locales)^42^, the reconstruction of specific cooking methods may also help to test the validity of modern analogues of shell midden formation from ethnographic or ethnohistoric observations^43^. Detailed information about specific processing techniques potentially broaden our understanding of prehistoric trading activities as the use of processing methods which prevent spoiling was most likely a prerequisite for the long-term transportation of the edible portion of marine molluscs^4^. Moreover, potential seasonal differences in shellfish processing techniques can contribute to our understanding of fishing and foraging seasonality. For long-term shell midden assemblages deposited over several millennia, the reconstruction of specific processing techniques may enable tracing the technological advancement over time of shell midden accretion.

Given the apparent alteration of shell δ^13^C values, potential alteration of ^14^C signatures and subsequent uncertainties in measured radiocarbon ages obtained from aragonitic shell midden constituents have to be expected. This hypothesis is supported by previous experiments showing that radiocarbon dates measured from food crusts on prehistoric pottery fragments can also be subjected to alteration during cooking by e.g. a reversed old-wood effect^44^. Detailed future studies are therefore needed to quantify the effect of pre-depositional heating on radiocarbon signatures in shell midden constituents.

## Material and Methods

### Modern Mercenaria campechiensis shells

In total, 23 sub-adult *M*. *campechiensis* individuals were cultured by *Southern Cross Sea Farms*®, at Cedar-Key, FL, USA (N29° 08′ 32.09662″ W83° 00′ 19.66703″). All individuals had the same age (14 months), similar size (~3.52 ± 0.24 cm shell height) and were reared in the same water masses throughout ontogeny. All individuals were sampled on 30 April 2015 and shipped overnight to RSMAS and sacrificed on 01 May 2015 immediately after arrival. Shells were manually cleaned with tap water, dried at room temperature and stored at ~20 °C until the experiment.

We used natural seawater from Virginia Key, FL, USA heated in a glass jar on a hotplate for the boiling treatment. Evaporation was compensated by adding periodically deionized water. A ~10 cm thick layer of commercial charcoal briquettes (Kingsford^®^ *Hickory*) were used to simulate roasting and burning. The coal briquettes were continually replenished to keep the burning and roasting temperature as constant as possible throughout the entire experiment. For the roasting treatment, individual shells were placed into perforated aluminium cups approximately 15 cm above the coal fire. For the burning treatment, individual shells were placed into small aluminium cups preventing any loss of shell material during the experiment or contamination and placed directly on/in between the burning coals. The shells were not buried in burning coal. The temporal and spatial temperature variability was determined for both treatments recurrently by measuring the temperature at different spots using an Omega^®^ HH806AU thermocouple thermometer equipped with an Uxcell^®^ K-type (nickel-chromium/nickel) high-temperature thermocouple probe (100–1250 °C). The reported treatment temperatures represent average values with the 1σ standard deviations. The measured treatment temperatures were boiling in seawater at ~100 °C, roasting over charcoal at ~174 ± 13 °C (maximum temperature range 126–205 °C) and burning on charcoal at 559 ± 95 °C (maximum temperature range 386–721 °C), see Supplemental Fig. 1. In each treatment, the right valves of six shells were exposed for 15 to 360 minutes while five untreated shells were used as control. Afterwards, all shells were cleaned for 24 hours with 60 ml ultrapure 10% hydrogen peroxide buffered to a pH of ~8.2 with NaOH following the protocol of Bastidas & Garcìa^45^ at room temperature to remove the periostracum and other organic compounds and ground using a ceramic pestle and mortar. Powdered bulk samples were stored in sealed glass vials at room temperature.

### Mid-Holocene samples

Mid-Holocene specimens were excavated from a shell midden in the Ras el Sass area, Mauritania, NW Africa. For details of the study area and spatial distribution of shell midden deposits see Barusseau *et al*.^46^ and the map provided in the supplementary information (Supplementary Fig. 3). All shells and otoliths were ultrasonically cleaned in deionised water and dried at 40 °C for 48 hours. Shells were cut along the direction of maximum growth and otoliths along the transversal plane using a tap water-cooled UniPrec WOCO 50 saw with a 0.6 mm thick diamond blade. The hinge region of each shell was sampled using a U-Power^®^ UP200 hand drill equipped with a 0.8 mm tungsten-carbide dental drill at lowest possible RPM (RPM ≈ 1000). Thick sections (~2 mm) were prepared along the direction of maximum growth and were sampled along the direction of growth in the outer shell layer using a NewWave/Merchantec Micromill system. Posterior halves of the shells and otoliths were ground using a ceramic pestle and mortar. Powdered samples were stored in sealed glass vials at room temperature until chemical analysis. The anterior halves of the otoliths were ground in a similar manner with ~50 mg aliquots used for mineralogical analyses and radiocarbon dating. Similarly, a piece of each shell was prepared for mineralogical analysis and radiocarbon dating by cutting a slice through the ventral margin of the anterior halves using a Dremel™ tool equipped with a diamond cutting wheel.

### Mineralogical analyses

Shell mineralogy of the experimental shells was measured using a PANalytical X´Pert PRO diffractometer with a Cu-tube (kα 1.541 Å, 45.0 kV, 40.0 mA) at RSMAS, Miami, FL, USA. Measurements were done using a continuous scan from 23.0–35.0 2θ with a step size of 0.005 2θ and a measuring time of 0.1 seconds per step. Data collection and processing was done using the software X’Pert Data Collector and X’Pert HighScore Plus, respectively. Relative abundance of aragonite and calcite were determined using the relative peak areas of aragonite (1,1,1) peak (2θ = 26.1°) and low magnesium calcite (1,0,1) peak (2θ = 29.6°) against a calibrated standard. Archaeological XRD-samples were analysed with a Philips X´Pert Pro diffractometer equipped with a Cu-tube (kα 1.541, 45 kV, 40.0 mA) at the University of Bremen, Germany. Measurements were done using a continuous scan from 3–85° 2θ with step sizes of 0.016° 2θ. We used the Philips software X´Pert HighScore™ for data processing. Abundance of aragonite and calcite was determined using the relative peak areas of aragonite (1,1,1) peak (2θ = 26.1°) and low magnesium calcite (1,0,1) peak (2θ = 29.6°) against a calibrated standard. Mineralogical data of the experimental and mid-Holocene samples are reported in the supplementary information (Supplementary Tab. 1 and 4).

### Radiocarbon dating

Radiocarbon dating of four otoliths and three bivalve shells was done at the Poznan Radiocarbon Laboratory, Poznan, Poland using a standard protocol for accelerator mass spectrometry. Calibration of conventional radiocarbon ages was done with OxCal 4.2.4^47^ using the Marine13 calibration curve^48^ and assuming a local reservoir age of ΔR = −300 yrs., which represents an average estimate for the local mid-Holocene reservoir ages based on several paired radiocarbon dates along the Mauritanian coastline throughout the Holocene (J. F. Saliége, unpublished data) which is also supported by radiocarbon dates from this archaeological site. Raw as well as calibrated radiocarbon ages are reported in the supplementary information (Supplementary Tab. 3).

### Clumped-isotope analysis

Clumped-isotope analysis of the powdered bulk samples was performed at the Stable Isotope Laboratory of the Rosenstiel School of Marine and Atmospheric Science (RSMAS), Miami, FL, United States, between April 17, 2015 and March 28, 2016 using the analytical procedure described in Murray *et al*.^49^. Each sample was analysed at least two times in a randomised order. All carbonate samples were prepared for analysis on the stainless-steel cryogenic vacuum extraction line at RSMAS, evacuated to <10^−6^ mbar using two turbo-molecular pumps (Balzer TPU 170 and Edwards 50EX). Individual sample aliquots of ~8.0 mg powder were digested for 30 minutes at 90 °C in 3.5 ml ~105% phosphoric acid (H_3_PO_4_) using a modified “Fairbanks” device^50^ connected to a common acid bath. Resulting CO_2_ was then purified on the cryogenic vacuum extraction line.

Purified CO_2_ was measured at RSMAS using a dual-inlet MAT-253 gas source isotope ratio mass spectrometer (Thermo Fisher Scientific, Bremen, Germany). Samples were measured against an in-house working-gas gas standard. We analysed each sample at a signal intensity of 12 V on mass-44 over six acquisitions of 15 sample-standard measurements. Data reduction and normalisation for ∆_47_ and temperature calculations followed the methods of Affek and Eiler^51^ and Huntington *et al*.^52^. To check for potential contamination, samples were scrutinised based on their offset of δ^48^ and ∆_48_^52^. Precision of clumped-isotope analysis is reported using the average standard errors. The external precision of the instrument was monitored with repeated measurement of a Carrara marble standard. The average Carrara marble ∆_47_ value was found to be 0.4001 ± 0.0294‰ (n = 191) which is similar to reported average values from four different laboratories of 0.395‰^24^ even though a different sample of the Carrara marble standard was used.

We measured δ^13^C and δ^18^O of the samples by means of masses 45/44 and 46/44, respectively using a method adapted from Craig^53^ modified for a multi-collector mass spectrometer. Shell and otolith δ^13^C and δ^18^O values were calibrated using NBS-19 and reported relative to Vienna Pee Dee Belemnite (VPDB). External precision of δ^13^C and δ^18^O analyses was better than ± 0.055 and ± 0.117‰, respectively. All Δ_47_ values defined as

$${{\rm{\Delta }}}_{47}=[(\frac{{R}^{47}}{{R}^{47}* }-1)-(\frac{{R}^{46}}{{R}^{46}* }-1)-(\frac{{R}^{45}}{{R}^{45}* }-1)]\,* \,1000$$

where R^47^, R^46^, R^45^ are abundance ratios of masses 47, 46 and 45 relative to the mass 44^25^, were calculated using the method described by Affek and Eiler^51^ and Huntington *et al*.^52^. Translation into the absolute reference frame (ARF) was accomplished using the method described by Dennis *et al*.^24^ with 1000, 50 and 25 °C water equilibrated gasses. All isotope data are reported in the supplementary information (Supplementary Tab. 1 and 4).

Recent publications by Schauer *et al*.^54^ and Daëron *et al*.^55^ have suggested a small difference between the correction protocols proposed by Santrock *et al*.^56^ and Brand *et al*.^57^ leading to differences in the calculated δ^13^C, δ^18^O and Δ_47_ values. These differences are small (~0.02‰) for δ^13^C and δ^18^O and generally within the external precision of the analytical method. For Δ_47_, the suggested changes were more significant and dependent upon the δ^13^C value of the material being analysed. These differences increased from essentially no changes for a sample with a δ^13^C value close to 0‰ to 0.04‰ for a sample with a δ^13^C value of −40‰. We compared our method of calculating δ^13^C and δ^18^O values modified from Craig^53^ and found the difference between our method and that proposed by Brand *et al*.^57^ to show only a 0.03‰ difference for samples with a δ^13^C difference of −40‰. This decreases to less than 0.01‰ for δ^13^C values of samples analysed in this study. Hence, based on the range of δ^13^C values of the samples measured in this study we conclude that the artefacts noted by Schauer *et al*.^54^ and Daeron *et al*.^55^ do not significantly impact the results presented here. We have, nonetheless, processed all results presented herein using the revised parameters from Brand *et al*.^55^.

### Ontogenetic oxygen-isotope records

Sub-samples drilled from the outer shell layer (~80–100 μg) were measured on a Finnigan MAT 251 gas isotope ratio mass spectrometer connected to a Kiel III automated carbonate preparation device at the stable isotope laboratory in the Center for Marine Environmental Sciences (MARUM), University of Bremen, Germany. The long-term standard deviation of the in-house standard (Solenhofen limestone) was better than 0.06‰ for δ^18^O.

### Statistical analysis

We used a one-way analysis of variances (ANOVA) with a Bonferroni-corrected t-test as post hoc test. The assessment of equal variances and normal distributions was done using a Levene’s test and a Shapiro-Wilk test, respectively. In case of non-normal distributed data (mineralogy), we used a Kruskal-Wallis one way ANOVA on ranks and the Dunn’s method as post hoc test. The significant levels for all statistical test were set to α = 0.05. Results of the statistical analyses are reported in the supplementary information (Supplementary Tab. 2).

### Data Availability

All data generated or analysed during this study are included in this published article (and its Supplementary Information files).

## Electronic supplementary material

Supplementary Information

## References

[1] Richards MP, Jacobi R, Cook J, Pettitt PB, Stringer CB (2005). Isotope evidence for the intensive use of marine foods by Late Upper Palaeolithic humans. J. Hum. Evol..

[2] Richards MP, Hedges REM (1999). Stable isotope evidence for similarities in the types of marine foods used by Late Mesolithic humans at sites along the Atlantic coast of Europe. J. Archaeol. Sci..

[3] Gordillo S, Brey T, Beyer K, Lomovasky BJ (2015). Climatic and environmental changes during the middle to late Holocene in southern South America: A sclerochronological approach using the bivalve *Retrotapes exalbidus* (Dillwyn) from the Beagle Channel. Quat. Int..

[4] Waselkov GA (1987). Shellfish gathering and shell midden archaeology. Adv. Archaeol. Method Therory.

[5] Andrus CFT (2011). Shell midden sclerochronology. Quat. Sci. Rev..

[6] Balbo A, Madella M, Godino IB, Álvarez M (2011). Shell midden research: An interdisciplinary agenda for the Quaternary and Social Sciences. Quat. Int.

[7] Disspain, M. C. F., Ulm, S. & Gillanders, B. M. Otoliths in archaeology: Methods, applications and future prospects. *J*. *Archaeol*. *Sci*. *Reports*, doi:10.1016/j.jasrep.2015.05.012 (2015).

[8] Sadler J (2012). Reconstructing past upwelling intensity and the seasonal dynamics of primary productivity along the Peruvian coastline from mollusk shell stable isotopes. Geochemistry, Geophys. Geosystems.

[9] Latorre, C., De Pol-Holz, R., Carter, C. & Santoro, C. M. Using archaeological shell middens as a proxy for past local coastal upwelling in northern Chile. *Quat*. *Int*. 1–9, doi:10.1016/j.quaint.2015.11.079 (2015).

[10] Takesue RK, van Geen A (2004). Mg/Ca, Sr/Ca, and stable isotopes in modern and Holocene Protothaca staminea shells from a northern California coastal upwelling region. Geochim. Cosmochim. Acta.

[11] Kim, J. S., Woo, K. S. & Hong, W. High resolution geochemical investigation of the bivalve shells (*Glycymeris* sp.) from shell mounds in Jeju Island, Korea: Late Holocene paleoclimatic implications related to East Asian Monsoon climate. *Quat*. *Int*. 1–10, doi:10.1016/j.quaint.2015.07.050 (2015).

[12] Azzoug M, Carré M, Schauer AJ (2012). Reconstructing the duration of the West African Monsoon season from growth patterns and isotopic signals of shells of *Anadara senilis* (Saloum Delta, Senegal). Palaeogeogr. Palaeoclimatol. Palaeoecol..

[13] Azzoug M (2012). Positive precipitation–evaporation budget from AD 460 to 1090 in the Saloum Delta (Senegal) indicated by mollusk oxygen isotopes. Glob. Planet. Change.

[14] Andrus CFT, Crowe DE, Sandweiss DH, Reitz EJ, Romanek CS (2002). Otolith δ^18^O record of mid-Holocene sea surface temperatures in Peru. Science.

[15] Wang T, Surge D, Walker KJ (2013). Seasonal climate change across the Roman Warm Period/Vandal Minimum Transition using isotope sclerochronology in archaeological shells and otoliths, Southwest Florida, USA. Quat. Int.

[16] Surge D, Walker KJ (2005). Oxygen isotope composition of modern and archaeological otoliths from the estuarine hardhead catfish (*Ariopsis felis*) and their potential to record low-latitude climate change. Palaeogeogr. Palaeoclimatol. Palaeoecol.

[17] Cohen AL, Parkington JE, Brundrit GB, van der Merwe NJ (1992). A Holocene marine climate record in mollusc shells from the Southwest African coast. Quat. Res..

[18] Carre M (2014). Holocene history of ENSO variance and asymmetry in the eastern tropical. Pacific. Science..

[19] Andrus CFT, Crowe DE (2002). Alteration of otolith aragonite: Effects of prehistoric cooking methods on otolith chemistry. J. Archaeol. Sci..

[20] Milano S, Prendergast AL, Schöne BR (2016). Effects of cooking on mollusk shell structure and chemistry: Implications for archeology and paleoenvironmental reconstruction. J. Archaeol. Sci. Reports.

[21] Larsen, S. C. Recrystallization of biogenic aragonite shells from archaeological contexts and implications for paleoenvironmental reconstruction. Master Thesis, Western Whasington University, WWU Master Thesis Collection. Paper 419 (2015).

[22] Schauble EA, Ghosh P, Eiler JM (2006). Preferential formation of ^13^C–^18^O bonds in carbonate minerals, estimated using first-principles lattice dynamics. Geochim. Cosmochim. Acta.

[23] Ghosh P (2006). ^13^C–^18^O bonds in carbonate minerals: A new kind of paleothermometer. Geochim. Cosmochim. Acta.

[24] Dennis KJ, Affek HP, Passey BH, Schrag DP, Eiler JM (2011). Defining an absolute reference frame for ‘clumped’ isotope studies of CO_2_. Geochim. Cosmochim. Acta.

[25] Eiler JM (2007). ‘Clumped-isotope’ geochemistry -The study of naturally-occurring, multiply-substituted isotopologues. Earth Planet. Sci. Lett..

[26] Dennis KJ, Schrag DP (2010). Clumped isotope thermometry of carbonatites as an indicator of diagenetic alteration. Geochim. Cosmochim. Acta.

[27] Huntington KW, Budd DA, Wernicke BP, Eiler JM (2011). Use of clumped-isotope thermometry to constrain the crystallization temperature of diagenetic calcite. J. Sediment. Res..

[28] Passey BH, Henkes GA (2012). Carbonate clumped isotope bond reordering and geospeedometry. Earth Planet. Sci. Lett..

[29] Staudigel PT, Swart PK (2016). Isotopic behavior during the aragonite-calcite transition: Implications for sample preparation and proxy interpretation. Chem. Geol..

[30] Grossman EL, Ku T-L (1986). Oxygen and carbon isotope fractionation in biogenic aragonite: Temperature effects. Chem. Geol..

[31] Lavaud R, Thébault J, Lorrain A, van der Geest M, Chauvaud L (2013). *Senilia senilis* (Linnaeus, 1758), a biogenic archive of environmental conditions on the Banc d’Arguin (Mauritania). J. Sea Res..

[32] Müller P (2015). Food for thought: Mathematical approaches for the conversion of high-resolution sclerochronological oxygen isotope records into sub-annually resolved time series. Palaeogeogr. Palaeoclimatol. Palaeoecol..

[33] Thorrold SR, Campana SE, Jones CM, Swart PK (1997). Factors determining δ^13^C and δ^18^O fractionation in aragonitic otoliths of marine fish. Geochim. Cosmochim. Acta.

[34] Wardecki D, Przeniosło R, Brunelli M (2008). Internal pressure in annealed biogenic aragonite. R. Soc. Chem..

[35] Stolper DA, Eiler JM (2015). The kinetics of solid-state isotope-exchange reactions for clumped isotopes: A study of inorganic calcites and apatites from natural and experimental samples. Am. J. Sci.

[36] Nouet J (2015). Limpet Shells from the Aterian Level 8 of El Harhoura 2 Cave (Témara, Morocco): Preservation State of Crossed-Foliated Layers. PLoS One.

[37] Loftus E, Rogers K, Lee-Thorp J (2015). A simple method to establish calcite:aragonite ratios in archaeological mollusc shells. J. Quat. Sci..

[38] Piasecki, A. Site-specific isotopes in small organic molecules. Dissertation, California Institue of Technology, Pasadena, CA, USA (2015).

[39] McNiven, I. J. & Wright, D. In *Terra Australis 29*; *Islands of inquiry*: *colonisation*,*seafaring and the archaeology of maritime landscapes* (eds Clark, G. & O’Connor, S.) 510 p. (ANU E Press, 2008).

[40] Thompson VD, Andrus CFT (2016). Evaluating mobility, monumentality, and feasting at the Sapelo Island shell ring complex. Am. Antiq..

[41] Makarewicz CA, Sealy J (2015). Dietary reconstruction, mobility, and the analysis of ancient skeletal tissues: Expanding the prospects of stable isotope research in archaeology. J. Archaeol. Sci..

[42] Bird DW, Bird RLB (1997). Contemporary shellfish gathering strategies among the Meriam of the Torres Strait islands, Australia: Testing predictions of a central place foraging model. J. Archaeol. Sci..

[43] Hardy K (2016). Shellfishing and shell midden construction in the Saloum Delta, Senegal. J. Anthropol. Archaeol..

[44] Philippsen, B. Variability of freshwater reservoir effects. Dissertation, Aarhus University (2012).

[45] Bastidas C, García E (1999). Metal content of the reef coral *Porites astreiodes*: An evaluation of river influence and 35 years of chronology. Mar. Pollut. Bull..

[46] Barusseau J-P, Vernet R, Saliège J-F, Descamps C (2007). Late Holocene sedimentary forcing and human settlements in the Jerf el Oustani - Ras el Sass region (Banc d’Arguin, Mauritania). Géomorphologie Reli. Process. Environ..

[47] Bronk Ramsey C (2009). Bayesian Analysis of Radiocarbon Dates. Radiocarbon.

[48] Reimer PJ (2013). Intcal13 and Marine13 radiocarbon age calibration curves 0–50,000 years cal BP. Radiocarbon.

[49] Murray ST, Arienzo MM, Swart PK (2016). Determining the Δ_47_ acid fractionation in dolomites. Geochim. Cosmochim. Acta.

[50] Swart PK, Burns SJ, Leder JJ (1991). Fractionation of the stable isotopes of oxygen and carbon in carbon dioxide during the reaction of calcite with phosphoric acid as a function of temperature and technique. Chem. Geol. Isot. Geosci. Sect..

[51] Affek HP, Eiler JM (2006). Abundance of mass 47 CO_2_ in urban air, car exhaust, and human breath. Geochim. Cosmochim. Acta.

[52] Huntington KW (2009). Methods and limitations of ‘clumped’ CO_2_ isotope (Δ_47_) analysis by gas-source isotope ratiomass spectrometry. J. Mass Spectrom..

[53] Craig H (1957). Isotopic standards for carbon and oxygen and correction factors for mass spectrometric analysis of carbon dioxide. Geochim. Cosmochim. Acta.

[54] Schauer, A. J., Kelson, J., Saenger, C. & Huntington, K. W. Choice of ^17^O correction affects clumped isotope (Δ_47_) values of CO_2_ measured with mass spectrometry. *Rapid Commun*. *Mass Spectrom*. (2016). 10.1002/rcm.7743 27650267

[55] Daëron M, Blamart D, Peral M, Affek HP (2016). Absolute isotopic abundance ratios and the accuracy of Δ_47_ measurements. Chem. Geol..

[56] Santrock J, Studley SA, Hayes JM (1985). Isotopic analyses based on the mass spectrum of carbon dioxide. Anal. Chem..

[57] Brand WA, Assonov SS, Coplen TB (2010). Correction for the ^17^O interference in δ(^13^C) measurements when analyzing CO_2_ with stable isotope mass spectrometry (IUPAC Technical Report). Pure Appl. Chem..

