# Supplementary material for: Prehistoric cooking versus accurate palaeotemperature records in shell midden constituents
Source: Sci Rep. 2017 Jun 15;7:3555. doi: 10.1038/s41598-017-03715-8 (PMC5472604; doi:10.1038/s41598-017-03715-8)
Supplement: Supplementary file 1 — Supplementary Information [file 41598_2017_3715_MOESM1_ESM.pdf]

Supplementary Information

Prehistoric cooking versus accurate palaeotemperature records in shell-midden constituents

Authors: Peter Müller, Philip T. Staudigel, Sean T. Murray, Robert Vernet, Jean-Paul Barusseau, Hildegard Westphal, Peter K. Swart

Page

Supplementary Figure 1: Treatment temperature data 2
Supplementary Data 1: Element/Ca ratio analysis.................... 3
Supplementary Figure 2: Impact of prehistoric cooking practises on shell element/Ca ratios.................... 4
Supplementary Figure 3: Map of the research area, Mauritania, NW Africa.................... 5
Supplementary Table 1: Mineralogy and proxy data of the cooking experiment. 6
Supplementary Table 2: Results of the statistical analysis.................... 7
Supplementary Table 3: Radiocarbon dates and calibrations using OxCal 4.2.4 8
Supplementary Table 4: Mineralogy and proxy data of the mid-Holocene samples 9
Supplementary References: 9

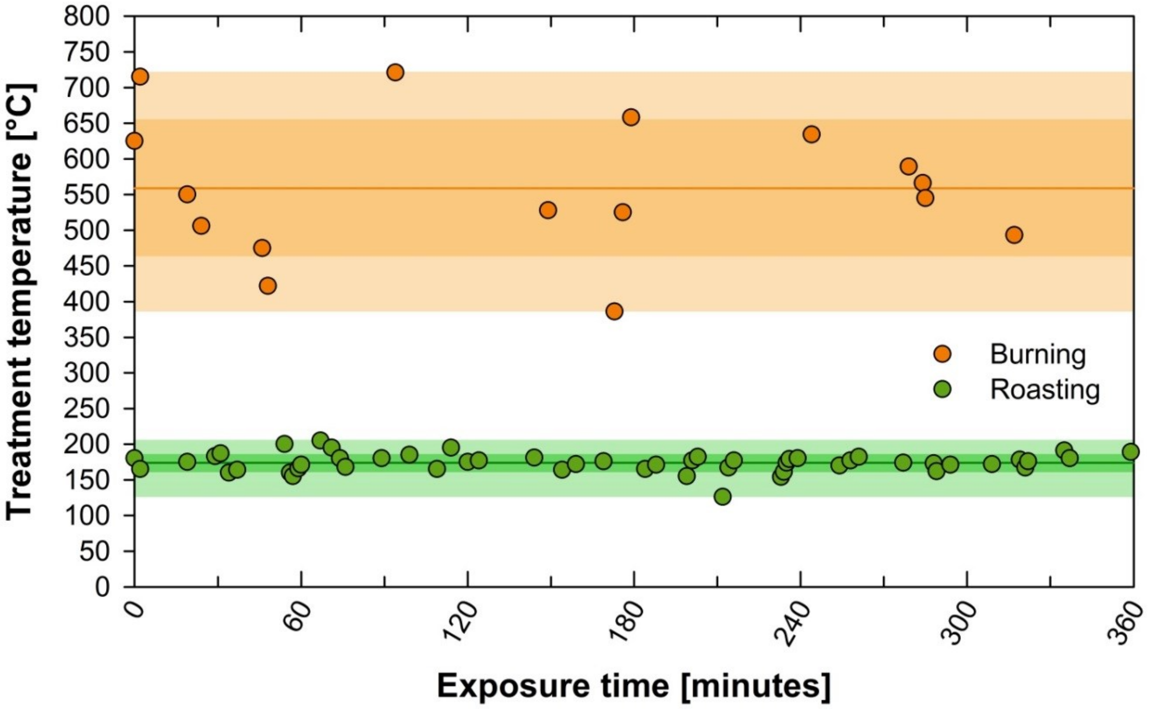

Supplementary Figure 1: Treatment temperatures measured using an Omega® HH806AU thermocouple thermometer equipped with an Uxcell® K-type (nickel-chromium/nickel) high-temperature thermocouple probe (100-1250 °C). Roasting temperatures were measured inside the perforated aluminium cups. The burning temperature was measured around as well as within the aluminium cups. The solid lines represent the calculated average temperature (174 and 559 °C). The dark shaded are is the calculated 1σ standard deviation of the treatment temperatures (±13 and ±95 °C) and the light shaded areas the maximum temperature range (126 – 205 °C and 386 – 721 °C for the roasting and burning treatment, respectively).

Supplementary Data 1: Element/Ca ratio analyses

To also assess the potential alteration of element/Ca ratios by prehistoric cooking practises, Mg/Ca, Sr/Ca and Ba/Ca ratios of all experimental shells were measured using a Spectro CIROS Vision inductively coupled plasma optical emission spectroscope (ICP-OES) at the Leibniz Centre for Tropical Marine Research (ZMT), Bremen, Germany. For each sample, ~ 0.5 g powdered material was dissolved in 20 ml supra pure 0.5 M nitric acid (Carl Roth®). Aliquots of 0.1 ml were further diluted with 0.5 M nitic acid. Instrument calibration solutions were prepared using single element standards in proportion to the *M. campechiensis* shell concentrations. Triplicate measurements of all shell samples were done routinely against the international reference standard JLs-1, an in-house working standard and nitric acid blanks. The accuracy for the triplicate measurements were < 4.18 %, < 3.03 %, < 0.26 %, and < 8.64 % for Ca, Mg, Sr and Ba, respectively with a precision of < 1 % for all elements. Measured element/Ca ratios are also reported in the Supplementary Tab. 1 and the results of the statistical analyses in Supplementary Tab. 2.

The measured element/Ca ratios also do not show a significant alteration, independent from treatment temperature and exposure time. Only Mg/Ca ratios of the boiled shells show a slight increase over time of exposure that can potentially be explained by the generally high Mg/Ca ratio of seawater (~5:1) used for the boiling treatment, favoring Mg/Ca substitution processes at the mineral surfaces. However, while the Mg/Ca ratios of all treatments tend towards higher values compared to the control shells, the range of Sr/Ca ratios measured in the treated shells exceeds that of the control shells indicating that substitution processes might also modify shell element/Ca ratios for some selected elements such as Mg or Sr.

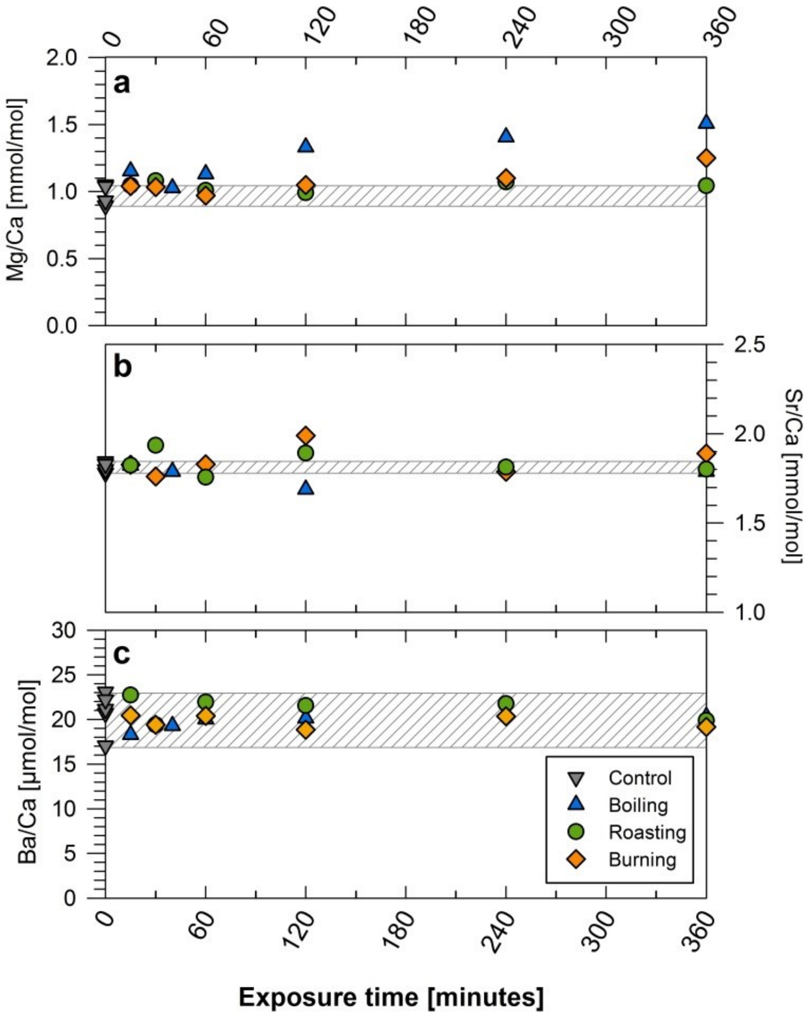

Supplemental Figure 2: Mg/Ca, Sr/Ca and Ba/Ca ratios of modern *Mercenaria campechiensis* shells exposed to the different prehistoric cooking practises. Shaded areas represent the range of measured element/Ca ratios of the control shells.

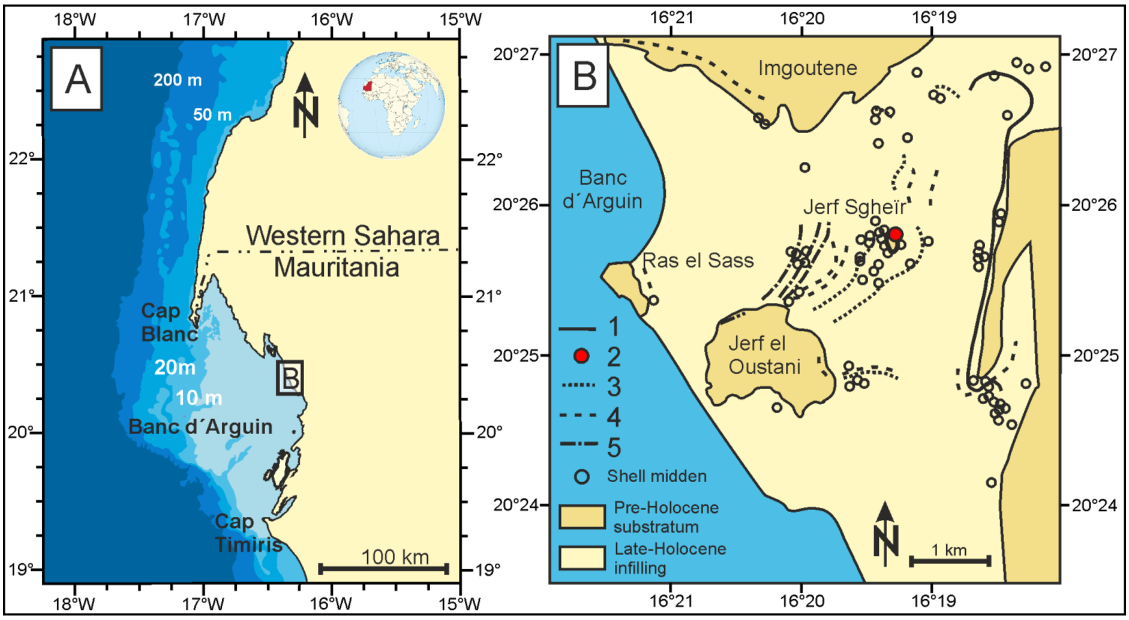

Supplementary Figure 3: Map of the research area, Mauritania, NW Africa: **A)** Mauritanian/Western Sahara coastline with the shallow shelf area of the Banc d´Arguin, modified from Müller et al.[1] with permission from Elsevier. **B)** Close-up of the study site in the hinterland of Ras el Sass in the mouth of a large paleo-estuary. The location of shell midden alignments and beach ridges show the human occupation pattern following the coastal progradation throughout the mid- to late-Holocene: 1: >5000 years BP according to archaeological evidences; 2: Jerf Sgheïr shell midden of this study (5,320-5,020 cal. yrs. BP); 3: Tintan group (4600-4000 uncal. yrs. BP); 4: Last Neolithic groups (3300 uncal. yrs. BP) 5: Copper Age groups, (2700-2600 uncal. yrs. BP), modified from Barusseau et al.[2] with permission from Géomorphologie : relief, processus, environnement; Revue du Groupe Français de Géomorphologie (GFG).

Supplementary Table 1: Mineralogy and proxy data of the cooking experiment. Each sample represents a single right valve of a *Mercenaria campechiensis* individual. Contamination of the initial aragonitic mineralogy within the control shells are related to the sporadic but clearly visible incorporation of dolomite grains within the inner shell layer originating from the dolomite catchment area of the adjacent estuaries.

Sample / Treatment	Exp. time [min]	Δ_{47} [‰, ARF]	SE (±) [‰, ARF]	$\delta^{18}O$ [‰, VPDB]	$\delta^{13}C$ [‰, VPDB]	Mg/Ca [mmol/mol]	Sr/Ca [mmol/mol]	Ba/Ca [µmol/mol]	Aragonite [wgt %]	Calcite [wgt %]	Dolomite [wgt %]
**Control**											
MC-1R	0	0.707	0.013	-0.19	-3.95	0.90	1.78	17.06	99.0	0.0	1.0
MC-2R	0	0.706	0.013	-0.19	-4.02	1.05	1.85	20.68	97.0	0.0	3.0
MC-3R	0	0.713	0.013	-0.35	-4.03	1.04	1.83	21.16	100.0	0.0	0.0
MC-4R	0	0.664	0.014	-0.30	-4.01	0.93	1.80	23.09	100.0	0.0	0.0
MC-5R	0	0.668	0.013	-0.33	-4.08	1.04	1.83	22.22	100.0	0.0	0.0
**Boiling**											
SW-15	15	0.674	0.016	-0.51	-4.16	1.15	1.82	18.29	100.0	0.0	0.0
SW-15	40	0.678	0.014	-0.88	-4.21	1.03	1.79	19.29	100.0	0.0	0.0
SW-15	60	0.685	0.014	-0.59	-3.98	1.13	1.78	20.03	100.0	0.0	0.0
SW-120	120	0.647	0.013	-0.49	-3.87	1.33	1.69	20.13	100.0	0.0	0.0
SW-240	240	0.667	0.012	-0.56	-3.90	1.41	1.81	20.75	100.0	0.0	0.0
SW-240	360	0.679	0.013	-0.76	-3.97	1.51	1.79	20.32	100.0	0.0	0.0
**Roasting**											
R-15R	15	0.605	0.013	-0.65	-4.09	1.05	1.82	22.72	100.0	0.0	0.0
R-30R	30	0.609	0.013	-0.72	-4.00	1.08	1.93	19.38	100.0	0.0	0.0
R-60R	60	0.542	0.014	-0.61	-3.91	1.01	1.76	21.91	100.0	0.0	0.0
R-120R	120	0.523	0.013	-0.77	-4.22	0.99	1.89	21.52	100.0	0.0	0.0
R-240R	240	0.510	0.014	-0.80	-4.04	1.07	1.81	21.74	100.0	0.0	0.0
R-360R	360	0.560	0.013	-0.50	-3.69	1.04	1.80	19.87	100.0	0.0	0.0
**Burning**											
B-15R	15	0.359	0.012	-0.87	-4.07	1.04	1.83	20.46	31.0	69.0	0.0
B-30R	30	0.390	0.013	-0.63	-3.97	1.03	1.76	19.41	83.0	17.0	0.0
B-60R	60	0.425	0.013	-0.73	-3.89	0.97	1.83	20.40	81.0	19.0	0.0
B-120R	120	0.415	0.011	-0.91	-4.30	1.05	1.99	18.84	100	0.0	0.0
B-240R	240	0.380	0.015	-0.95	-4.04	1.10	1.79	20.35	31.0	69.0	0.0
B-360R	360	0.342	0.013	-1.17	-4.35	1.25	1.89	19.15	0.0	100.0	0.0

Supplementary Table 2: Results of the statistical analysis using a one-way ANOVA with a Bonferroni-corrected t-test as post hoc test. The assessment of equal variances and normal distributions was done using a Levene's test and a Shapiro-Wilk test, respectively. For data with unequal variances or non-normal distribution, we used a Kruskal-Wallis one way ANOVA on ranks and the Dunn's method as post hoc test to test. The significant levels for all statistical test were set to $\alpha = 0.05$. Bold numbers indicate significant differences between two treatments.

**Treatments**	Δ_{47}	$\delta^{18}O$	$\delta^{13}C$	**Mg/Ca**	**Sr/Ca**	**Ba/Ca**	**Mineralogy***
Control vs. boiling	>0.05	**<0.05**	>0.05	**<0.05**	>0.05	>0.05	>0.05
Control vs. roasting	**<0.001**	**<0.001**	>0.05	>0.05	>0.05	>0.05	>0.05
Control vs. burning	**<0.001**	**<0.001**	>0.05	>0.05	>0.05	>0.05	>0.05
Boiling vs. roasting	**<0.001**	>0.05	>0.05	>0.05	>0.05	>0.05	>0.05
Boiling vs. burning	**<0.001**	**<0.05**	>0.05	>0.05	>0.05	>0.05	**<0.05**
Roasting vs. burning	**<0.001**	>0.05	>0.05	>0.05	>0.05	>0.05	**<0.05**

*) ANOVA on ranks

Supplementary Table 3: Results of the radiocarbon dating and calibration using OxCal 4.2.4. Radiocarbon dates of three bivalve shells and four otoliths excavated from the Ras el Sass area, Mauritania, NW Africa. Radiocarbon ages were calibrated with OxCal 4.2.4 [3] using the Marine13 calibration curve [4] with a local ΔR value of -300 yrs. (J.-F. Saliège, unpublished data) which is supported by the analysis of paired terrestrial/marine samples (Ash-1/OTO-BX), providing a local estimate for the ΔR value of ~-330 yrs.

Sample	Material	Lab Code	^{14}C age [yrs BP]	Uncertainty [^{14}C yrs]	ΔR [yrs.]	cal. yrs. BP from	cal. yrs. BP to	Median	Uncertainty + [yrs]	Uncertainty - [yrs]
**Layer 1**										
JS-SS-1	Bivalve	Poz-69422	4830	35	-300	5270	4990	5130	140	140
JS-SS-2	Bivalve	Poz-69424	4770	35	-300	5210	4880	5020	190	140
JS-SS-3	Bivalve	Poz-69425	4845	35	-300	5280	5020	5150	130	130
**Layer 2**										
Ash-1	Ashy soil	Pa2344	4900	40						
OTO-B3	Otolith	Poz-60777	4920	30	-300	5330	5070	5260	70	190
OTO-B4	Otolith	Poz-60778	4970	30	-300	5430	5250	5320	110	70
OTO-B8	Otolith	Poz-71188	4920	35	-300	5390	5060	5260	130	200
OTO-B9	Otolith	Poz-71189	4970	35	-300	5440	5240	5320	120	80

Supplementary Table 4: Mineralogy and proxy data of the mid-Holocene samples. Clumped isotopic composition, conventional oxygen and carbon isotope as well as mineralogical raw data of the mid-Holocene samples excavated from a shell midden at the northern Mauritanian coast, NW Africa. Minor occurrences of primary calcite within catfish otoliths from Mauritania was also found in modern fish otoliths (<2 % calcite).

Sample	Organism	Sample type	Δ_{47} [‰, ARF]	SE (±) [‰, ARF]	$\delta^{18}O$ [‰, VPDB]	$\delta^{13}C$ [‰, VPDB]	Aragonite [wgt %]	Calcite [wgt %]
**Shell midden layer 1**								
JS-SS-1	Bivalve	Bulk	0.666	0.014	0.61	-0.28	100.0	0.0
	Bivalve	Hinge	0.651	0.016	0.88	-0.50	-	-
JS-SS-2	Bivalve	Bulk	0.601	0.014	0.10	-0.66	100.0	0.0
	Bivalve	Hinge	0.667	0.014	-0.15	-1.01	-	-
JS-SS-3	Bivalve	Bulk	0.667	0.013	0.50	0.22	100.0	0.0
	Bivalve	Hinge	0.691	0.015	0.65	-0.11	-	-
JS-SS-4	Bivalve	Bulk	0.631	0.012	-0.23	-0.04	100.0	0.0
	Bivalve	Hinge	0.686	0.014	-0.12	-0.33	-	-
JS-SS-5	Bivalve	Bulk	0.661	0.013	-0.54	-0.11	100.0	0.0
	Bivalve	Hinge	0.683	0.014	-0.27	-0.43	-	-
JS-SS-6	Bivalve	Bulk	0.716	0.013	0.44	-0.02	100.0	0.0
	Bivalve	Hinge	0.676	0.014	0.21	0.50	-	-
JS-SS-10	Bivalve	Bulk	0.622	0.017	0.26	0.38	100.0	0.0
	Bivalve	Hinge	0.697	0.015	0.55	-0.02	-	-
**Shell midden layer 2**								
OTO-B3	Otolith	Bulk	0.716	0.013	-0.19	-1.57	98.0	2.0
OTO-B4	Otolith	Bulk	0.714	0.012	0.03	-0.73	96.0	4.0
OTO-B8	Otolith	Bulk	0.704	0.014	0.05	-0.89	100.0	0.0
OTO-B9	Otolith	Bulk	0.667	0.016	-0.11	-1.92	100.0	0.0

Supplementary References:

1. Müller, P. *et al.* Food for thought : Mathematical approaches for the conversion of high-resolution sclerochronological oxygen isotope records into sub-annually resolved time series. *Palaeogeogr. Palaeoclimatol. Palaeoecol.* 440, 763–776 (2015).

2. Barusseau, J.-P., Vernet, R., Saliège, J.-F. & Descamps, C. Late Holocene sedimentary forcing and human settlements in the Jerf el Oustani - Ras el Sass region (Banc d'Arguin, Mauritania). *Géomorphologie Reli. Process. Environ.* 7, 7–18 (2007).

3. Bronk Ramsey, C. Radiocarbon calibration and analysis of stratigraphy: The OxCal program. *Radiocarbon* 37, 425–430 (1995).

4. Reimer, P. J. *et al.* Intcal13 and Marine13 radiocarbon age calibration curves 0 – 50,000 years cal BP. *Radiocarbon* 55, 1869–1887 (2013).
